# Global disease burden of breast cancer attributable to high fasting plasma glucose: a comprehensive analysis from the global burden of disease study

**DOI:** 10.3389/fendo.2025.1498207

**Published:** 2025-02-13

**Authors:** Jing Zhang, Jiawei He, Yunyan Lu, Tian Lan

**Affiliations:** ^1^ Department of Cardiology, The First People’s Hospital of Xiaoshan District, Xiaoshan Affiliated Hospital of Wenzhou Medical University, Hangzhou, Zhejiang, China; ^2^ Department of Breast Surgery, Hangzhou TCM Hospital Affiliated to Zhejiang Chinese Medical University, Hangzhou Hospital of Traditional Chinese Medicine, Hangzhou, Zhejiang, China

**Keywords:** breast cancer, high fasting plasma glucose, global disease burden, mortality, disability-adjusted life years

## Abstract

**Background:**

High fasting plasma glucose (HFPG) has been identified as one of the risk factors associated with the development of breast cancer. The worldwide distribution of breast cancer attributable to HFPG was not comprehensively investigated.

**Methods:**

We utilized the data from the Global Burden of Disease Study 2021 to explore HFPG-related breast cancer deaths, disability adjusted life years (DALYs) and corresponding age-standardized rates (ASRs). The average annual percentage change (AAPC) and the estimated annual percentage change (EAPC) were employed to evaluate the temporal trend.

**Results:**

The global effect of HFPG resulted in nearly 30,570 breast cancer deaths and 819,550 DALYs in 2021, representing an age-standardized deaths rate (ASMR) of 0.66 (95% UI -0.19-1.57) and an age-standardized DALYs rate (ASDR) of 18.05 (95% UI -5.31-42.71). In the regions with low, low-middle, and middle SDI, the ASRs of HFPG-related breast cancer increased significantly over time. The highest ASMR and ASDR were observed in several countries, such as Palau, American Samoa, Cook Islands, Marshall Islands, and United Arab Emirates. There was a positive correlation between ASRs and Socio-Demographic Index (SDI) in countries where SDI was below 0.75. The escalation in death and DALYs was primarily driven by epidemiological change and population growth in low, low-middle, middle SDI regions.

**Conclusions:**

Substantial disparities exist across diverse regions in breast cancer burden attributed to HFPG. It is urgent to regulate glycemic levels, improve healthcare infrastructures, and provide cost-effective care in less developed and developing countries that endure a disproportionately heavier health burden.

## Introduction

Breast cancer is the leading cause of cancer-related deaths in women, resulting in 2,308,897 new cases and 665,684 deaths in 2022 (1). Several metabolic factors, such as obesity, metabolic syndrome and type 2 diabetes, have been proved to be associated with an increased risk of developing several types of cancers, including breast cancer (2–4). The prevalence of type 2 diabetes has risen dramatically for decades, as overweight, sedentary lifestyle and population aging, which significantly jeopardize the prognosis of breast cancer patients (5).

High fasting plasma glucose (HFPG) is an abnormal metabolic state, which has been indubitably evidenced to harbor correlations with various malignancies (6–8). Previous researches revealed that the insulin-associated pathways, estrogen fluctuations, cytokine aberrations, and Warburg effect could potentially underpin the interrelation between HFPG and breast cancer (9–12). Diabetes could detrimentally influence both locoregional management and overall survival in patients with advanced breast cancer (13). Another study demonstrated that hyperglycemia led to an increase in breast cancer mortality and metastasis (14).

Previous evidence has suggested that HFPG can contribute to an increased global burden of several diseases, such as bladder cancer, stroke, and ischemic heart disease (15–17). The global burden of breast cancer was explored in some researches (18, 19). However, there is no meticulous and specific appraisal of HFPG-related breast cancer burden. In light of the substantial surge in the HFPG prevalence over the past three decades, it is imperative to evaluate the impact of HFPG on breast cancer burden. The most recent iteration of the Global Burden of Disease (GBD) study 2021 presents as a fundamental resource for evaluating the global cancer burden attributable to various risk factors. In this study, we conducted an analysis at the global, regional, and national levels to appraise the trends in breast cancer burden attributable to HFPG from 1990 to 2021, utilizing data derived from the GBD study 2021, in order to obtain the relevant information on the epidemiological trend and to ascertain the preferential direction for investing medical resources.

## Methods

### Data sources and collection

The GBD study 2021 provided an exhaustive and comparative evaluation of 288 causes of death and 88 risk factors, thereby enhancing our current understanding of global health challenges (20). The scope of the study encompassed 21 regions and 204 countries or territories spanning the period from 1990 to 2021. In our study, we retrieved data pertaining to mortality and disability-adjusted life years (DALYs), along with age-standardized rates (ASRs) of breast cancer attributed to HFPG, from the GBD Study 2021 (http://ghdx.healthdata.org/gbd-results-tool). DALYs were computed by amalgamating the years of life lost and the years lived with disability. ASRs were utilized to evaluate and compare the deaths and DALYs rates among regions and countries with distinct demographic characteristics and age structures. According to average educational attainment, fertility rate, and economic condition, sociodemographic index (SDI) was estimated for each countries or territories, ranging from 0 (worst) to 1 (best). Based on SDI in 2021, 204 countries or territories were classified into five distinct categories: low, low-middle, middle, high-middle, and high SDI groups. The human development index (HDI) among countries or territories were sourced from the United Nations Human Development Report (http://hdr.undp.org/en/data). In addition, the GBD study 2021 provided accurate elucidation for HFPG and delineated the Theoretical Minimum-Risk Exposure Level (TMREL). The HFPG was characterized as any measurement exceeding the TMREL of 4.8-5.4 mmol/L (21, 22). Following the GBD methodology, the estimations were adjusted for other relevant risk factors and their potential interactions with HFPG.

### Statistical analyses

In assessing the chronological trajectories, we employed dual methodologies encompassing the average annual percentage change (AAPC) and the estimated annual percentage change (EAPC). The AAPC, inclusive of the corresponding 95% confidence interval (CI), was evaluated by the joinpoint analysis. The detailed formula was set as followed,


$$AAPC=\left\{exp\left(\frac{\sum^{}w_{i}b_{i}}{\sum^{}w_{i}}\right)\right\}\times 100$$

where *b* signified the coefficient for the segment, *wi*​ indicated the length of the segment, and *i* pertained to the specific *i*th segment. Additionally, EAPC was calculated by a linear regression, two detailed equations were established as followed.


ln(ASRs)= α + βX + ϵ

EAPC = 100 × (exp(β) – 1)

In these formulae, X signified the calendrical year, ϵ embodied the error component, while β designated the coefficient.

Decomposition analysis, motivated by Bashir’s and Das Gupta’s method, was applied to gain insights into the disparities in disease burden across different regions (23). This approach attributed DALYs difference to the combined influences of three factors, such as population size, population aging, and epidemiological changes. We calculated the contributions of these three components by using Cheng’s decomposition method (24). The association between ASRs and SDI was evaluated by the Pearson’s correlation analysis. We utilized the R package “BACP” to forecast the disease burden from 2022 to 2030, informed by the Global Population Forecasts data and existing ASRs (19). All statistical calculations and visual representations were accomplished through the R program (version 4.1.2). The determination of statistical significance was predicated upon a P value beneath 0.05.

## Results

### Global breast cancer burden caused by HFPG

**Figure 1:**
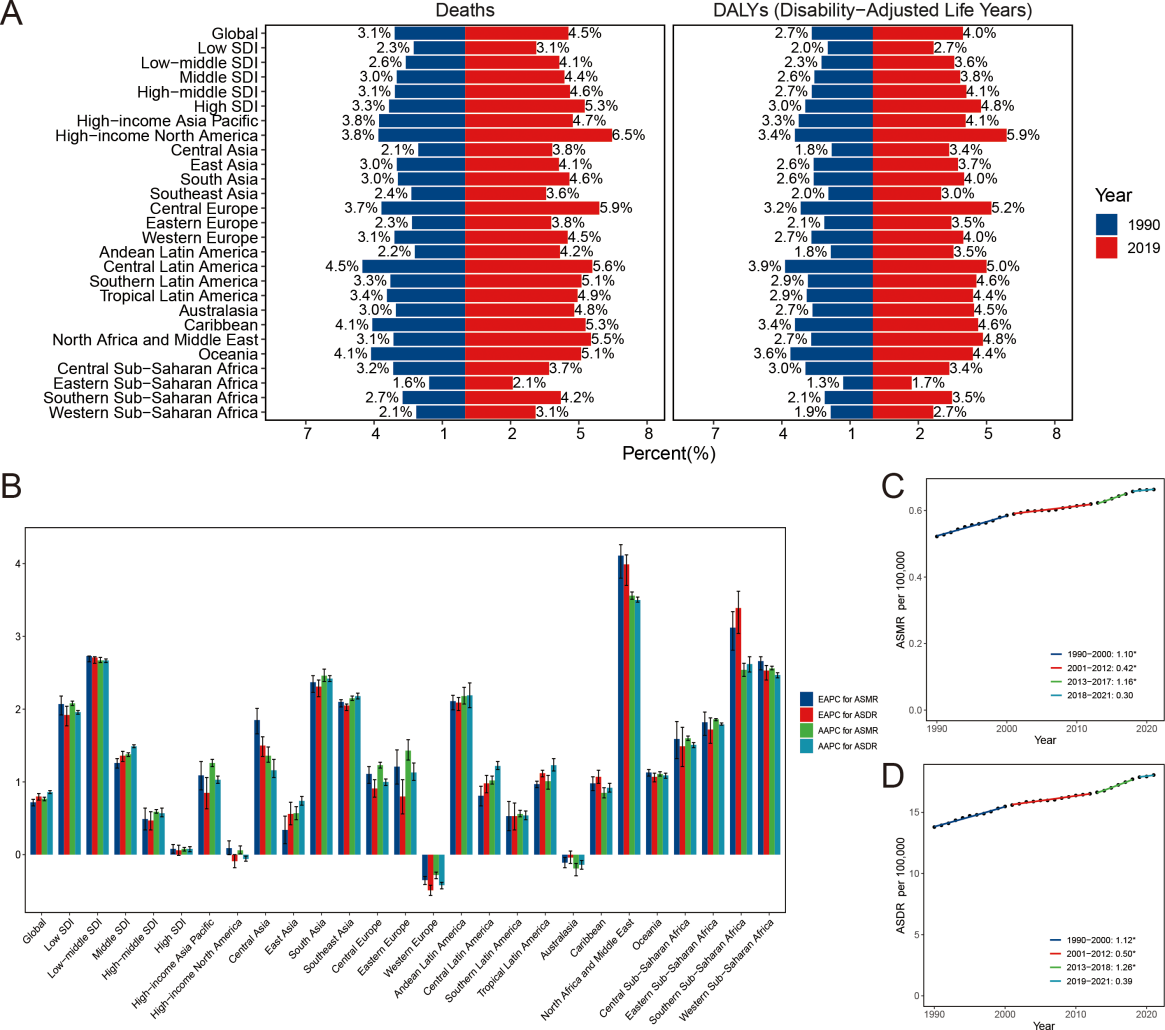
The proportion of breast cancer burden due to high fasting plasma glucose in 1990 and 2021 **(A)**. Temporal change of breast cancer due to high fasting plasma glucose **(B)**. The global APCs in the age-standardized mortality **(C)** and DALYs **(D)** rate for breast cancer attributable to high fasting plasma glucose. *P<0.05, APC, annual percentage change; DALYs, disability-adjusted life-years; EAPC, estimated annual percentage change; ASMR, age-standardized mortality rate; ASDR, age-standardized DALYs rate.

In 2021, approximately 4.5% of breast cancer-related fatalities and 4.0% of DALYs were estimated to be attributed to HFPG, aggravating the overall burden of the disease (**Figure 1A**). The global impact of HFPG resulted in approximately 31 thousand breast cancer deaths and 0.82 million DALYs, representing an ASMR of 0.66 (95% UI -0.19 -1.57) per 100,000 population and an ASDR of 18.05 (95% UI -5.31-42.71) per 100,000 population (**Table 1**, **Supplementary Table S1**). The ASRs in 2021 were higher than those recorded in 1990, suggesting an upward trend as indicated by positive values for both EAPC (0.72, 95%CI: 0.67-0.76 for ASMR; 0.80, 95%CI: 0.75-0.84 for ASDR) and AAPC (0.77, 95%CI: 0.74-0.79 for ASMR; 0.86, 95%CI: 0.84-0.88 for ASDR) (**Table 1**, **Supplementary Tables S1**, **S2**, **Figure 1B**, **Supplementary Figure S1**). It revealed three periods of continuous increase in the ASMR and ASDR based on the joinpoint analysis (**Figures 1C, D**).

**Table 1:** The global DALY burden of breast cancer attributable to high fasting plasma glucose and the relevant temporal trend.

Characteristics	DALY in 1990 No. × 10^3^ (95% UI)	ASDR in 1990 No. (95%UI)	DALY in 2019 No. × 10^3^ (95% UI)	ASDR in 2019 No. (95%UI)	EAPC 1990-2019 No. (95%CI)
Global	484.39 (88.94-1094.21)	22.70 (4.18-51.14)	1239.76 (237.91-2787.94)	28.46 (5.46-64.02)	0.82 (0.78-0.86)
Low SDI	19.24 (3.45-45.71)	15.19 (2.71-36.39)	83.55 (15.85-196.71)	29.22 (5.59-67.89)	2.21 (2.14-2.23)
Low-middle SDI	54.63 (9.60-128.68)	16.92 (3.05-40.06)	236.18 (43.72-551.05)	31.61 (5.89-73.58)	2.01 (1.90-2.09)
Middle SDI	92.01 (17.07-212.66)	16.52 (3.10-37.83)	333.08 (63.86-746.86)	24.48 (4.71-54.75)	1.28 (1.22-1.32)
High-middle SDI	129.89 (24.23-294.42)	21.63 (4.03-49.12)	271.89 (52.35-606.96)	24.69 (4.75-55.28)	0.44 (0.27-0.61)
High SDI	188.23 (35.77-419.60)	32.97 (6.20-73.86)	313.92 (61.45-685.97)	33.38 (6.46-73.26)	0.28 (0.10-0.46)
High-income Asia Pacific	9.62 (1.74-22.45)	8.55 (1.55-19.95)	24.45 (4.63-55.89)	12.45 (2.33-28.66)	1.06 (0.98-1.13)
High-income North America	89.06 (16.92-198.28)	48.24 (9.08-107.82)	141.55 (28.00-307.56)	43.90 (8.66-95.81)	0.46 (0.08-0.83)
Central Asia	5.86 (1.09-13.48)	21.09 (3.91-48.59)	15.63 (2.90-35.18)	34.65 (6.49-77.50)	2.08 (1.87-2.24)
East Asia	57.21 (10.55-132.79)	12.06 (2.23-27.92)	154.41 (27.97-362.83)	13.87 (2.51-32.67)	0.56 (0.35-0.76)
South Asia	50.62 (8.78-120.25)	16.95 (3.00-40.18)	257.00 (48.22-600.13)	34.10 (6.42-79.39)	2.22 (2.10-2.30)
Southeast Asia	30.91 (5.68-74.58)	20.92 (3.88-49.81)	108.75 (20.09-248.93)	31.44 (5.84-71.67)	1.06 (0.84-1.26)
Central Europe	23.67 (4.39-52.92)	28.48 (5.28-63.87)	42.57 (8.48-97.79)	36.81 (7.26-85.32)	0.98 (0.90-1.05)
Eastern Europe	26.10 (4.75-60.17)	15.42 (2.79-35.83)	39.64 (7.46-91.11)	19.73 (3.68-45.63)	0.43 (0.11-0.76)
Western Europe	113.29 (21.73-253.85)	35.51 (6.71-79.77)	171.09 (34.71-374.19)	37.11 (7.46-82.48)	-0.04 (-0.09- 0.01)
Andean Latin America	1.32 (0.25-3.04)	12.20 (2.31-28.18)	5.87 (1.18-13.70)	19.99 (4.02-46.58)	1.56 (1.44-1.65)
Central Latin America	11.92 (2.33-26.19)	26.53 (5.25-57.80)	44.41 (8.83-101.22)	34.19 (6.83-77.86)	0.80 (0.72-0.87)
Southern Latin America	8.18 (1.56-18.66)	31.86 (6.05-72.83)	18.87 (3.60-41.83)	41.38 (7.83-91.85)	0.65 (0.50-0.81)
Tropical Latin America	14.05 (2.67-31.83)	27.83 (5.31-62.78)	35.28 (6.57-79.45)	26.30 (4.90-59.20)	-0.09 (-0.24- 0.06)
Australasia	2.65 (0.49-6.15)	21.47 (3.92-49.76)	5.91 (1.14-13.37)	23.66 (4.52-54.08)	-0.10 (-0.31- 0.12)
Caribbean	5.02 (0.99-11.31)	37.07 (7.29-83.28)	13.97 (2.92-31.17)	51.31 (10.67-114.50)	1.20 (1.13-1.27)
North Africa and Middle East	15.80 (2.80-37.47)	17.07 (3.03-40.16)	84.91 (17.38-192.84)	36.52 (7.39-82.08)	3.00 (2.81-3.10)
Oceania	1.10 (0.20-2.64)	63.13 (11.81-150.05)	5.75 (1.11-13.52)	133.51 (26.36-310.68)	2.72 (2.60-2.76)
Central Sub-Saharan Africa	2.37 (0.44-5.67)	18.50 (3.55-43.13)	9.78 (1.72-23.83)	31.75 (5.70-76.28)	1.83 (1.70-1.93)
Eastern Sub-Saharan Africa	5.12 (0.88-12.40)	12.71 (2.16-30.10)	16.41 (2.94-38.50)	18.45 (3.36-42.57)	1.23 (1.14-1.30)
Southern Sub-Saharan Africa	3.80 (0.72-8.79)	24.38 (4.61-56.21)	14.65 (2.83-32.94)	44.62 (8.64-99.43)	2.52 (2.15-2.83)
Western Sub-Saharan Africa	6.71 (1.17-16.02)	15.42 (2.71-36.69)	28.85 (5.24-68.67)	28.54 (5.26-67.76)	2.09 (1.97-2.17)

DALYs, disability-adjusted life years; UI, uncertainty interval; ASDR, age-standardized DALY rate; EAPC, estimated annual percentage change; CI, confidence interval; SDI, Socio-demographic Index.

### Regional breast cancer burden caused by HFPG

Regarding the SDI regions, HFPG had a pronounced impact on the breast cancer burden across all five SDI regions. In low, low-middle, and middle SDI regions, the ASRs of HFPG -related breast cancer increased significantly over time, supported by positive values for both AAPC and EAPC (**Table 1**, **Supplementary Tables S1**, **S2**, **Supplementary Figure S1**). It represented a stable pattern of the ASRs in high and high-middle SDI regions. During 2018-2021, there was a downward trend of ASRs in high SDI region (APC, -0.63, 95%CI: -1.24–0.23 for ASMR; APC, -0.52, 95%CI: -1.17–0.10 for ASDR) (**Supplementary Tables S3**, **S4**). However, ASDR was stable in high-middle SDI region during 2003-2021.

Regarding the GBD regions, East Asia, South Asia, Western Europe, and High-income North America suffered the highest breast cancer death and DALYs cases attributable to HFPG in 2021 (**Table 1**, **Supplementary Table S1**). While Oceania, Caribbean, Central Europe, and South Sub-Saharan Africa experienced the highest ASMR and ASDR. North Africa and Middle East and Southern Sub-Saharan Africa were top two regions with highest EAPC of ASRs. According to the AAPC value, Western Europe and Australasia were the only regions that showed a decreasing trend in ASRs, while the other regions displayed an increasing trend (**Supplementary Table S2**).

### National breast cancer burden caused by HFPG

**Figure 2:**
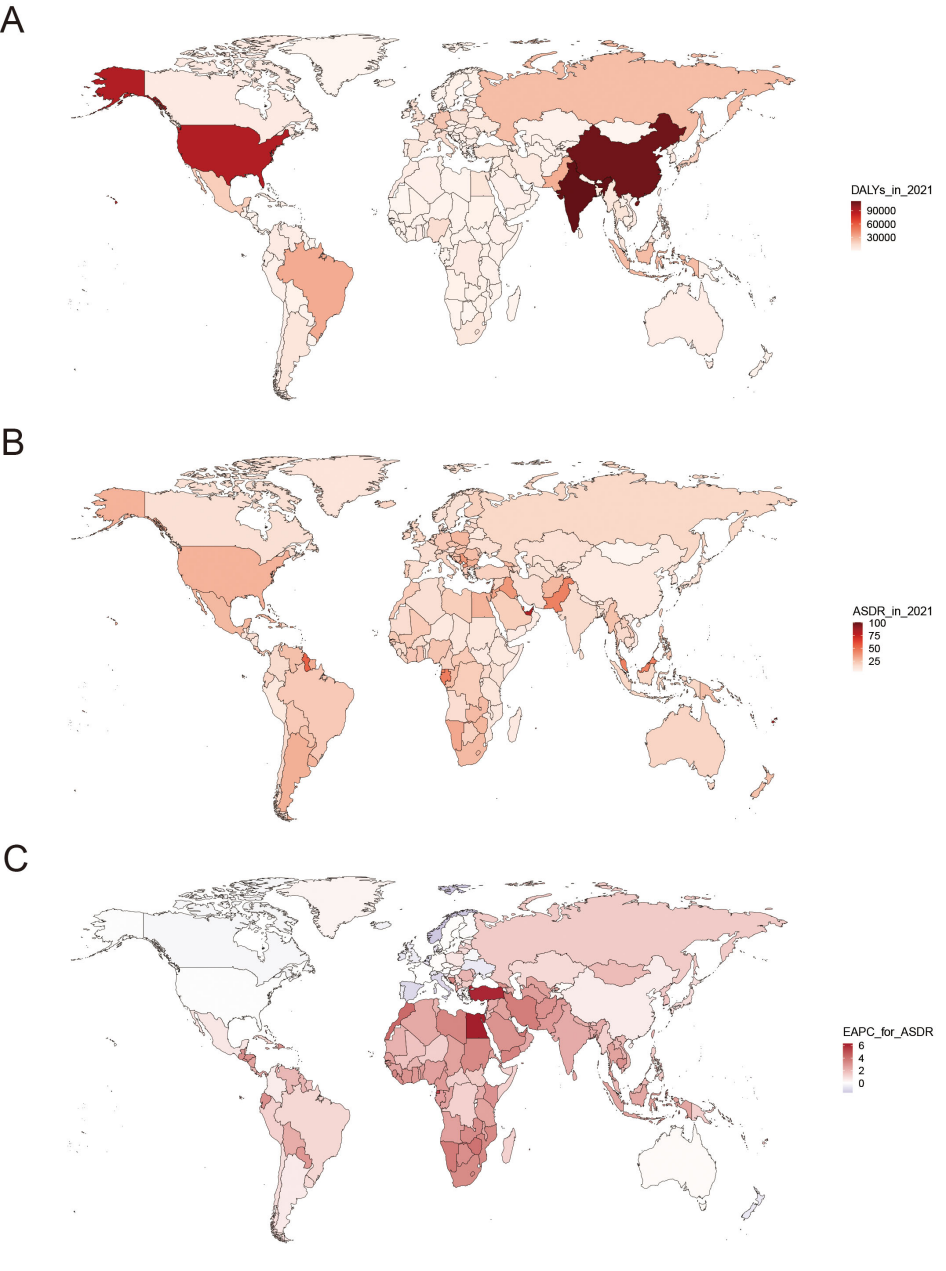
The global distribution of DALYs **(A)**, ASDR **(B)**, and corresponding EAPC **(C)** for breast cancer due to high fasting plasma glucose. DALYs, disability-adjusted life-years; ASDR, age-standardized DALYs rate; EAPC, estimated annual percentage change.

Regarding the national level, the burden of HFPG-related breast cancer displayed heterogeneity across 204 countries. Both ASMR and ASDR showed a downward trend in 18 and 20 countries, respectively. An upward trend was recorded for ASMR in 174 countries, and for ASDR in 167 countries (**Supplementary Table S5**). India, United States of America, and China were ranked as the top three countries with the highest number of death and DALYs cases (**Figure 2A**, **Supplementary Figure S2A**). The highest ASMR and ASDR were observed in several countries, such as American Samoa, Palau, Marshall Islands, Cook Islands, Fiji, Nauru, Tuvalu (Oceania), Qatar, Bahrain, United Arab Emirates (Middle East) (**Figure 2B**, **Supplementary Figure S2B**, **Supplementary Table S5**). Egypt, Turkey, Lesotho, and Equatorial Guinea had the highest EAPC value for both ASMR and ASDR, both exceeding five (**Figure 2C**, **Supplementary Figure S2C**). On the contrary, the lowest EAPC for both ASMR and ASDR were observed in Norway, Spain, Netherlands, Italy, and Bermuda.

### Global breast cancer burden attributable to HFPG by age and SDI

**Figure 3:**
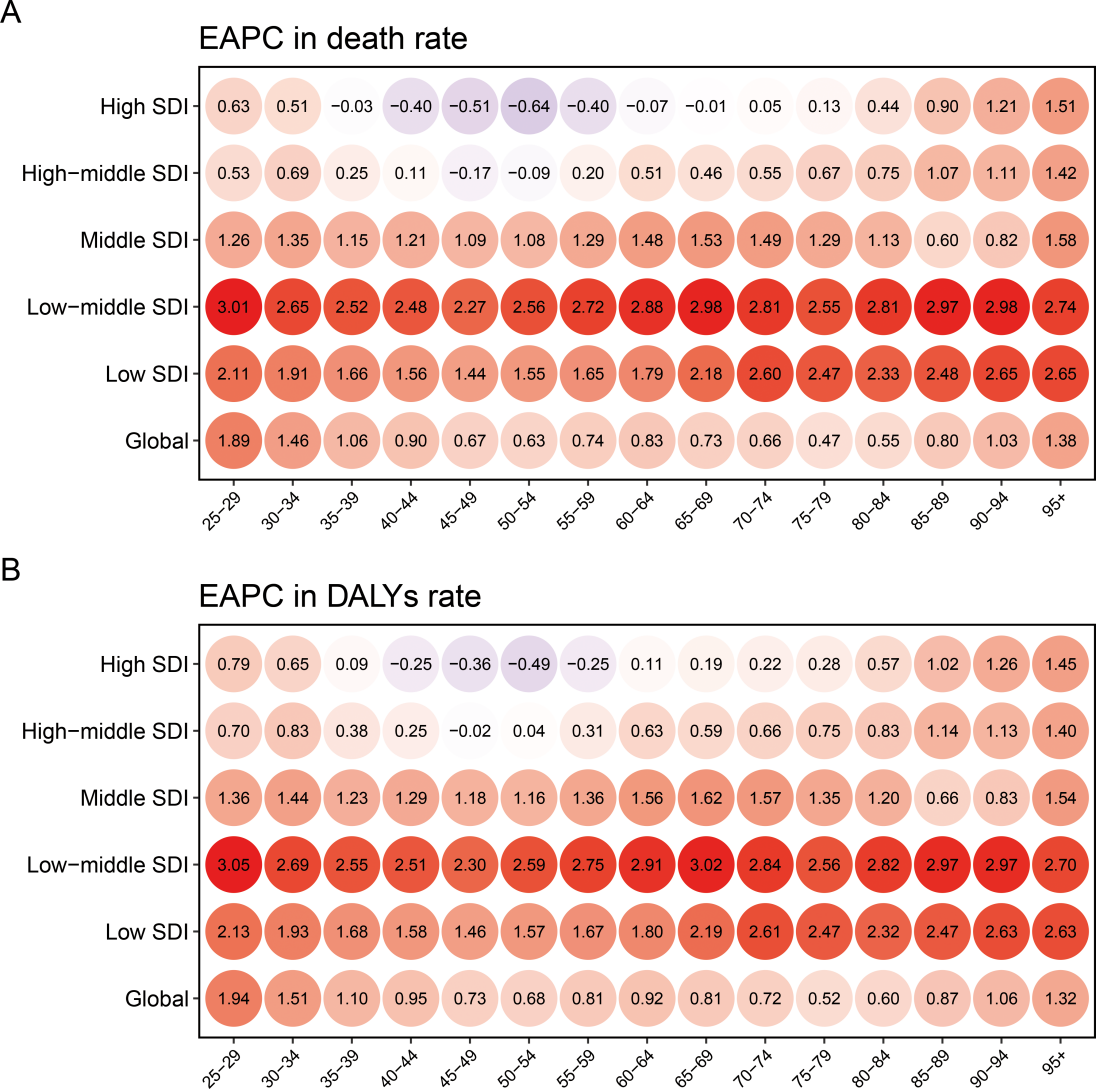
The EAPC of age specific death **(A)** and DALYs **(B)** rate for breast cancer due to high fasting plasma glucose, categorized by SDI, during 1990-2021 EAPC, estimated annual percent change; DALY, disability adjusted life-year; SDI, socio-demographic index.

Our finding presented an inverted-V shape for the death and DALYs cases (**Supplementary Figures S3A, C**). The peak of the inverted-V shaped trend was observed within the age group of 65-69 for deaths, and within the age group of 55-59 for DALYs. The age-specific breast cancer death rate associated with HFPG escalated with age growth (**Supplementary Figure S3B**). The age-specific DALYs rate increased among individuals younger than 65 and those older than 85, and remained stable in those aged 65-85 (**Supplementary Figure S3D**). Furthermore, the EAPCs of death rates exhibited positive values across all age groups in middle, low-middle, and low SDI regions for breast cancer attributable to HFPG, signifying a notable increase trend (**Figure 3A**). There was a declining trend of age-specific death rate in individual aged 45-55 in high-middle and high SDI regions. The change in age-specific DALYs rate shared similar pattern to age-specific death rate across various SDI regions (**Figure 3B**).

In the GBD regions with an SDI below 0.4, including Oceania, Eastern Sub-Saharan Africa, and Western Sub-Saharan Africa, a positive correlation was identified between ASRs and SDI (**Supplementary Figure S4**). It demonstrated an inverse V-shape relationship between ASRs and SDI in High-income North America, Australasia and Southern Latin America. There existed a positive correlation between ASRs and SDI in countries where SDI was below 0.75. Conversely, there was an inverse relationship observed in countries where SDI exceeded 0.75 (**Supplementary Figures S5A, B**). A negative relationship was observed between EAPCs and HDI in 2022 (R = -0.52, p < 0.01 for ASMR; R=-0.53, p<0.01 for ASDR);, which was particularly prominent in countries with higher HDI, but not in countries with an HDI below 0.7 (**Supplementary Figures S5C, D**).

### The decomposition of the change in breast cancer due to HFPG

**Figure 4:**
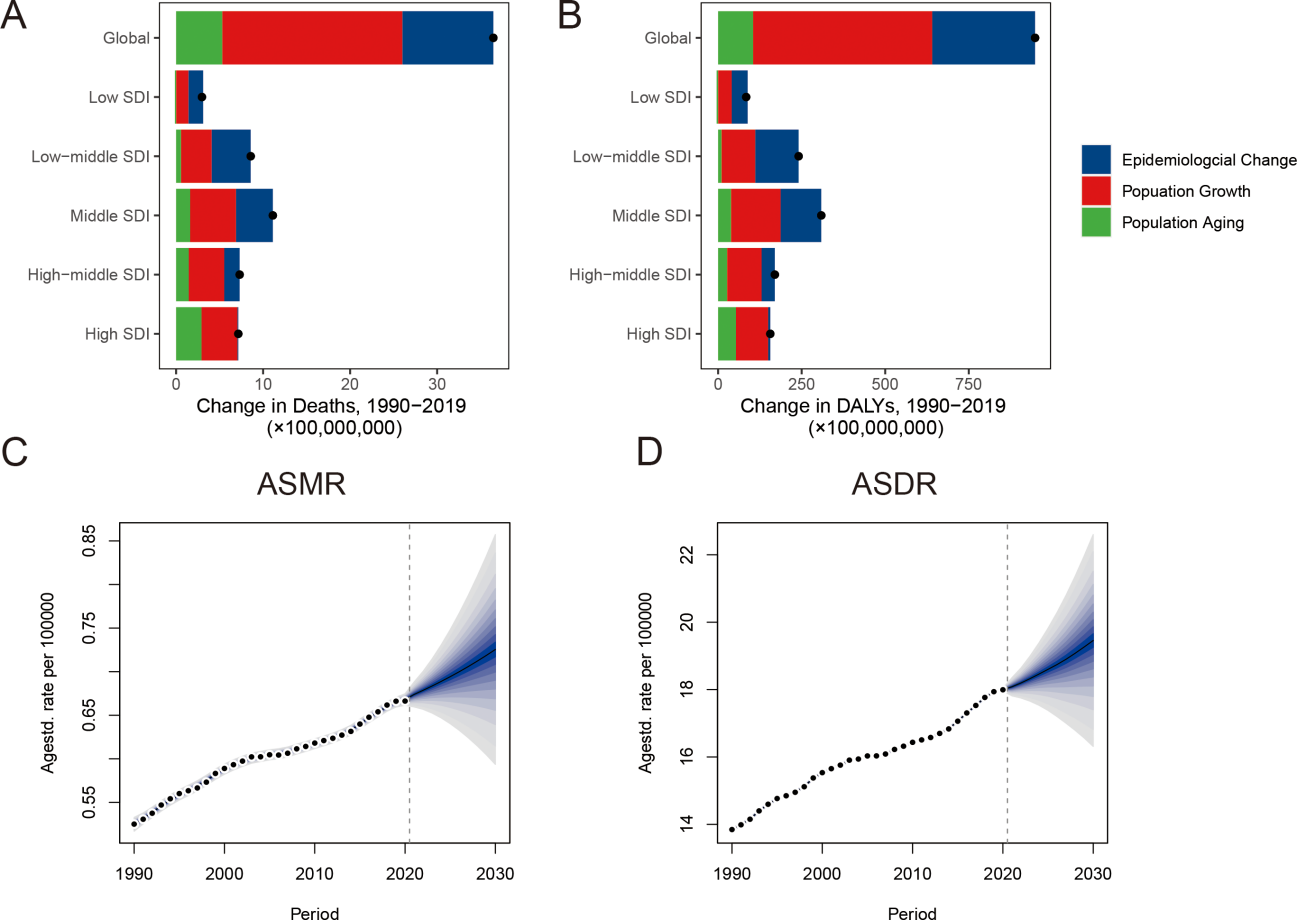
Decomposition analysis and future forecasts of breast cancer attributable to high fasting plasma glucose The change in death **(A)** and DALYs **(B)** of disease by decomposition analysis. The temporal trends of ASMR **(C)** and ASDR **(D)** from 1990 to 2030. The black dots present the overall alteration ascribed to population aging, population growth, and epidemiological change. ASMR, age-standardized mortality rate; ASDR, age-standardized DALYs rate.

It presented a substantial worldwide escalation in death and DALYs for breast cancer due to HFPG (**Figure 4**, **Supplementary Table S6**). The influence of all three population-level determinants on disease burden growth showed a positive effect. The escalation in death and DALYs for breast cancer due to HFPG was primarily driven by epidemiological change and population growth in low, low-middle, middle SDI regions. In contrast, it was primarily influenced by population aging and growth in high-middle and high SDI regions.

### Future forecasts of the breast cancer burden due to HFPG

We presented the future forecasts of the disease burden from 2021 to 2030 (**Figures 4C, D**). It was estimated that ASMR will increase from 0.53 per 100,000 population in 1990 to 0.73 per 100,000 population in 2030. There will be 30,830 death cases in 2030. Simultaneously, an upward trend of ASDR was anticipated in the future. The DALYs count was predicted to rise to 824,344 cases by 2030.

## Discussion

In this study, we investigated the global, regional, and national spatiotemporal trends of breast cancer attributable to HFPG over the period of 1990–2021. HFPG-related breast cancer continues to contribute significantly to the overall burden of breast cancer, encompassing substantial proportions of both mortality and DALYs. We also delved into the impact of age and SDI on HFPG-related breast cancer. A discernible upward trajectory was observed in ASMR and ASDR of breast cancer attributable to HFPG globally. The ASR trends displayed notable variations across different geographical and SDI regions. At the same time, we found that the burden of HFPG-related breast cancer was associated with SDI and age. The inverted-V shape observed in death and DALY cases may be partially elucidated by the age structure of the global population. Epidemiological change and population growth were the two major drivers of the escalation in death and DALYs in low, low-middle, middle SDI regions.

In the GBD study 2021, the definition of HFPG included not only individuals diagnosed with diabetes but also those with prediabetes (22). Individuals with obesity and type 2 diabetes were at greater risk of breast cancer relapse and mortality (25). In addition, an elevated risk of type 2 diabetes was observed in breast cancer survivors who treated with chemotherapy and tamoxifen (26). According to findings from a mendelian randomization study, diabetes was found to have a causal association with poorer breast cancer-specific survival (27). Moreover, the study suggested that treating diabetes could potentially enhance prognosis in individuals with breast cancer. To facilitate early detection and intervention among individuals at high risk of diabetes, the 1-h plasma glucose during a 75-gram oral glucose tolerance test may be a more sensitive and practical method, which could offer significant benefits to the sizeable population of breast cancer survivors (28). Healthful diet, weight management, and physical activity should be promoted to enhance the outcome of breast cancer survivors.

HFPG was reported to be associated with the development, recurrence, metastasis, and treatment of breast cancer. Hyperinsulinemia, hyperglycemia, and chronic inflammation were proposed to explain the relationship between HFPG and breast cancer (29). First, hyperinsulinemia was closely linked with insulin resistance. Insulin-like growth factor (IGF) receptors were commonly expressed on the membrane of cancer cells, where they could interact with their ligand to simulate cell proliferation, invasion, and metastasis and enhance cancer progression (30). Insulin had the ability to augment cellular metabolism, which can induce oxidative stress and DNA damage, consequently promoting oncogenesis (31). Second, hyperglycemia exacerbated DNA damage and impaired DNA repair mechanisms in breast cancer by activating the reactive oxygen species (ROS) pathway (32). Hyperglycemia and hyperinsulinemia exerted synergistic effects on breast cancer cell proliferation and migration by activating Akt and phospholipase C-dependent signal pathway (33). Third, inflammation was a key factor in the relationship between cancer and HFPG. Diabetes and hyperglycemia could alter the composition of gut microbiome, damage the integrity of intestinal barrier, and elevate oxidative stress levels, a key characteristic of chronic inflammation (34, 35). Moreover, hyperglycemia exposure triggered chronic low-grade inflammation by TNF-α signaling (32, 36). These inflammatory microenvironment caused by hyperglycemia eventually facilitates the proliferation, invasion, and metastasis of cancer cell.

We revealed a conspicuous regional and national disparity in the disease burden of HFPG-related breast cancer. A significant surge in ASRs was observed across most geographic regions, especially North Africa and Middle East and Southern Sub-Saharan Africa. These escalations may partially stem from high diabetes prevalence, poor glucose control, detrimental lifestyle habits, poor dietary practices, and environmental pollution (37–39). Besides, substantial increased prediabetes prevalence may be another reason (40). Oncologists, nurse practitioners, and patients ought to foster enhanced collaboration in patient care for the effective management of chronic conditions during cancer treatment (41). Metformin emerged as a potential therapeutic drug for the dual purpose of controlling blood glucose and treating breast cancer (42). Simultaneously, our study revealed a significant escalation in the burden of HFPG-related breast cancer across all SDI regions. The ASRs increased significantly during 1990-2021 across all age subgroups in the regions with low-middle and low SDI. Diminished health awareness, insufficient medical resources, and subpar healthcare quality may contribute to an amplified disease burden in the low SDI region (43–45). The pervasive negative sentiment stemming from unaffordable insulin prices may potentially trigger a rise of disease burden in high SDI region (46). The genomic studies should be performed in the future to understand potential mechanism underlying cross-country inequalities. Governments and policy makers should devise innovative global strategies according to region or country-specific circumstances.

There are several limitations inherent in this study. First, it is crucial to recognize that the availability of data sources was not exhaustive in the GBD study 2021. The absence of population-based cancer registries in certain countries significantly hampered the availability and reliability of crucial data required for estimating the burden of cancer. However, the updated Bayesian algorithm and non-zero floor method were helpful to reduce stochastic variation and estimate the relatively accurate data in the GBD study 2021 (47). Second, breast cancer is a complex and heterogeneous disease. Due to the absence of comprehensive clinicopathological information, we were unable to account for these confounding factors in investigating the global burden of HFPG-related breast cancer. Third, our analysis of the burden of HFPG-related breast cancer was conducted at global, regional, and national levels. We did not delve into a granular examination of local attributes, including variations between rural and urban areas.

## Conclusions

The burden of HFPG-related breast cancer in 2021 were higher than that recorded in 1990, revealing an upward trend of disease burden in nearly all regions. Marked differences were observed in the disease burden across distinct regions. The urgent need to control glycemic levels, bolster healthcare systems, and ensure affordable care is particularly palpable in less developed and developing countries that shoulder a disproportionately large health-associated burden. Given the rising global prevalence of diabetes, it is important to develop and implement comprehensive intervention strategies to diminish HFPG-induced breast cancer.

## Data Availability

Publicly available datasets were analyzed in this study. This data can be found here: http://ghdx.healthdata.org/gbd-results-tool, http://hdr.undp.org/en/data.

## Supplementary material

Supplementary Figure 1 ASMR and ASDR of breast cancer attributable to high fasting plasma glucose across various SDI regions during 1990-2021. ASMR, age-standardized mortality rate; ASDR, age-standardized DALY rate; SDI, socio-demographic index.

Supplementary Figure 2 The worldwide distribution of death **(A)**, ASMR **(B)**, and relevant EAPC **(C)** for breast cancer due to high fasting plasma glucose. ASMR, age-standardized mortality rate; EAPC, estimated annual percentage change.

Supplementary Figure 3 Breast cancer death **(A)** and DALYs **(C)** attributable to high fasting plasma glucose by age and SDI. The distribution of death **(B)** and DALYs **(D)** rate attributed to high fasting plasma glucose-related breast cancer by age. The grey area indicates the 95% uncertainty interval for the rate. DALYs, disability-adjusted life-years; SDI, socio-demographic index.

Supplementary Figure 4 The relationship between SDI and age-standardized rates (ASMR and ASDR) across 21 Global Burden of Disease regions. ASMR, age-standardized death rate; ASDR, age-standardized DALYs rate.

Supplementary Figure 5 The association between SDI and age-standardized rates for high fasting plasma glucose-related breast cancer across 204 countries or territories **(A, B)**. The relationship between HDI and EAPCs of age-standardized rates for high fasting plasma glucose-related breast cancer **(C, D)**. SDI, socio-demographic index; ASMR: age-standardized mortality rate; ASDR, age-standardized DALYs rate; EAPC, estimated annual percentage change; HDI, human development index.
