# Supplementary figures and images for: Global disease burden of breast cancer attributable to high fasting plasma glucose: a comprehensive analysis from the global burden of disease study
Source: Front Endocrinol (Lausanne). 2025 Feb 13;16:1498207. doi: 10.3389/fendo.2025.1498207 (PMC11864957; doi:10.3389/fendo.2025.1498207)

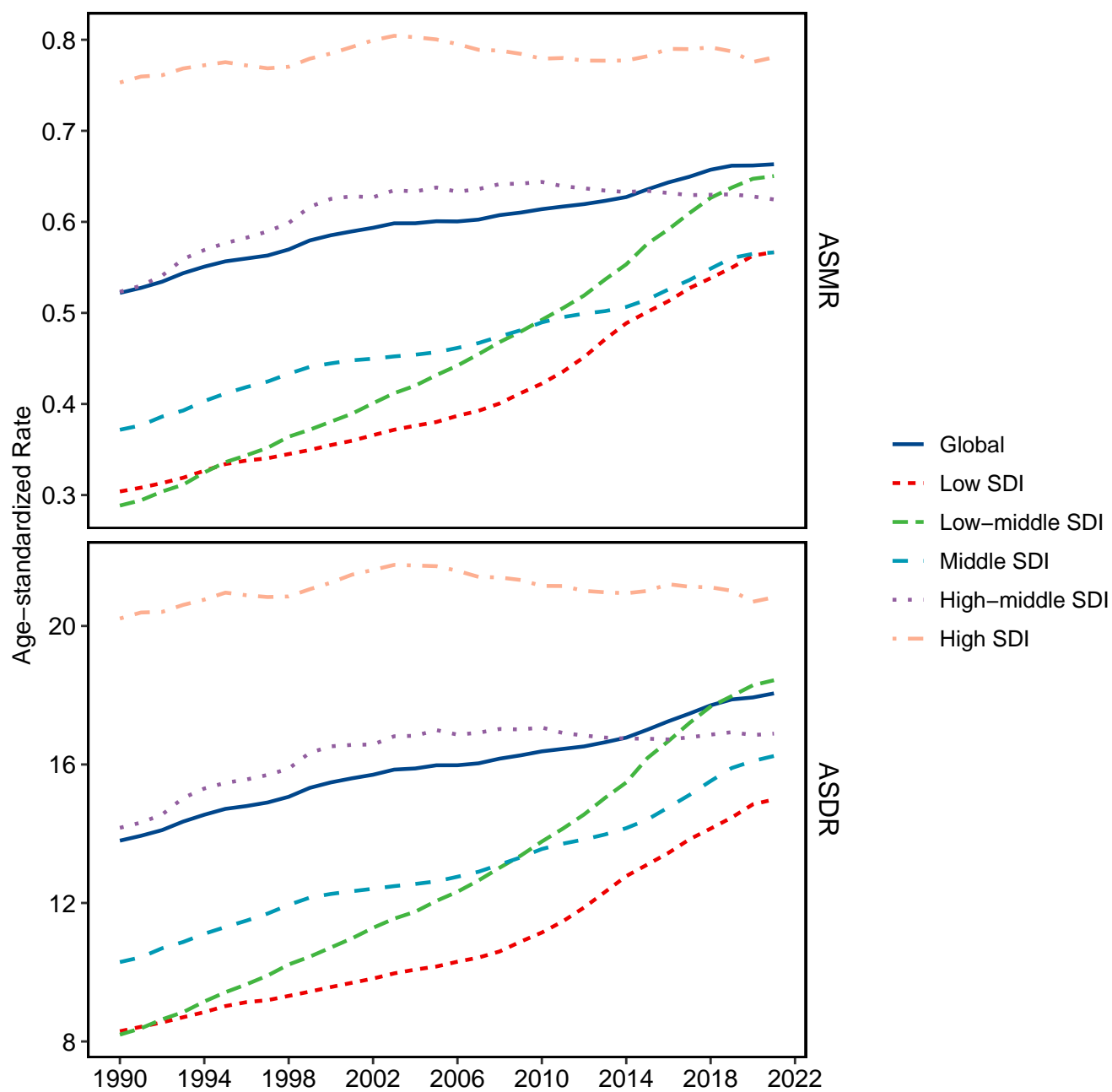

Supplement: Supplementary Figure 1 — ASMR and ASDR of breast cancer attributable to high fasting plasma glucose across various SDI regions during 1990-2021. ASMR, age-standardized mortality rate; ASDR, age-standardized DALY rate; SDI, socio-demographic index. [file Image1.pdf]

A

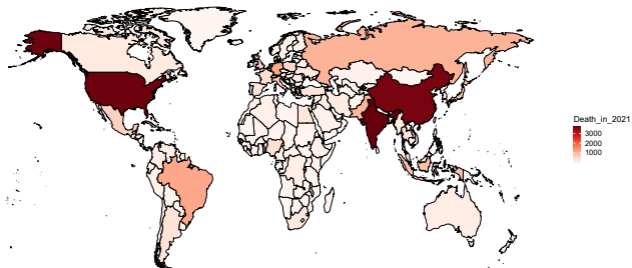

B

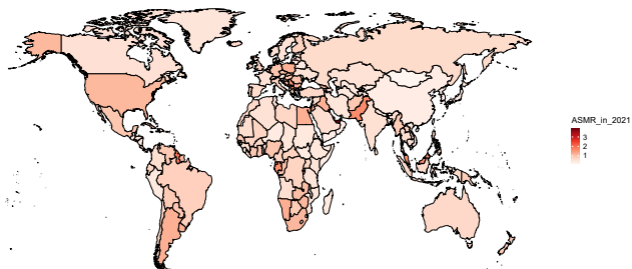

C

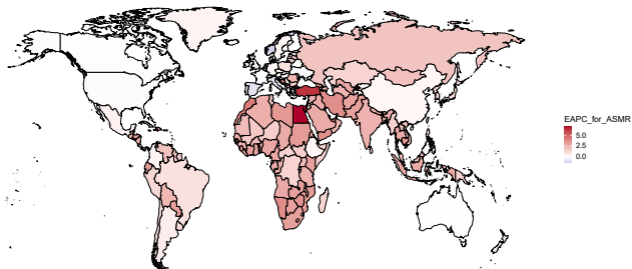

Supplement: Supplementary Figure 2 — The worldwide distribution of death (A), ASMR (B), and relevant EAPC (C) for breast cancer due to high fasting plasma glucose. ASMR, age-standardized mortality rate; EAPC, estimated annual percentage change. [file Image2.pdf]

A

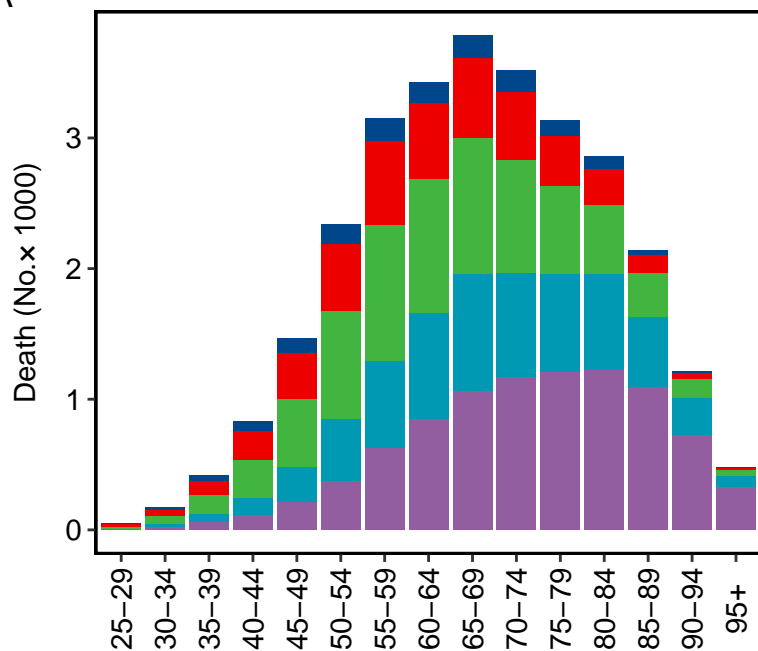

B

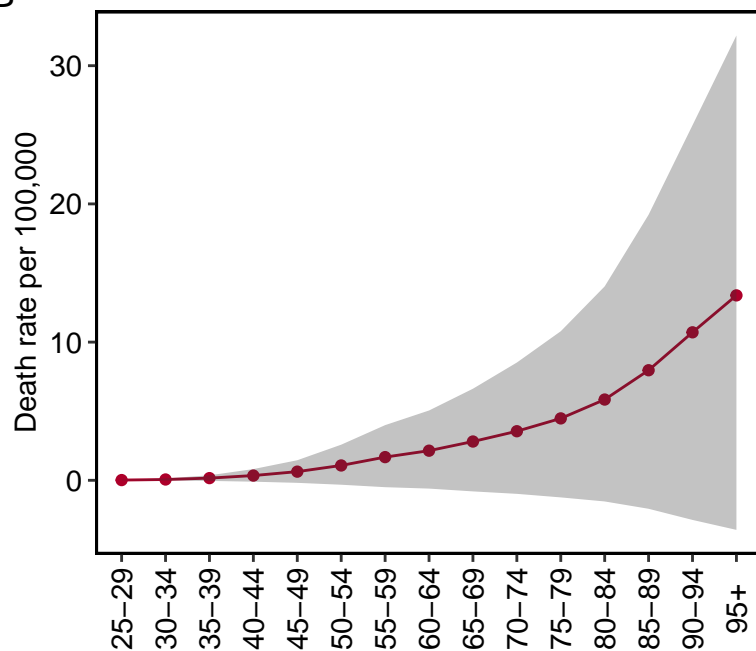

C

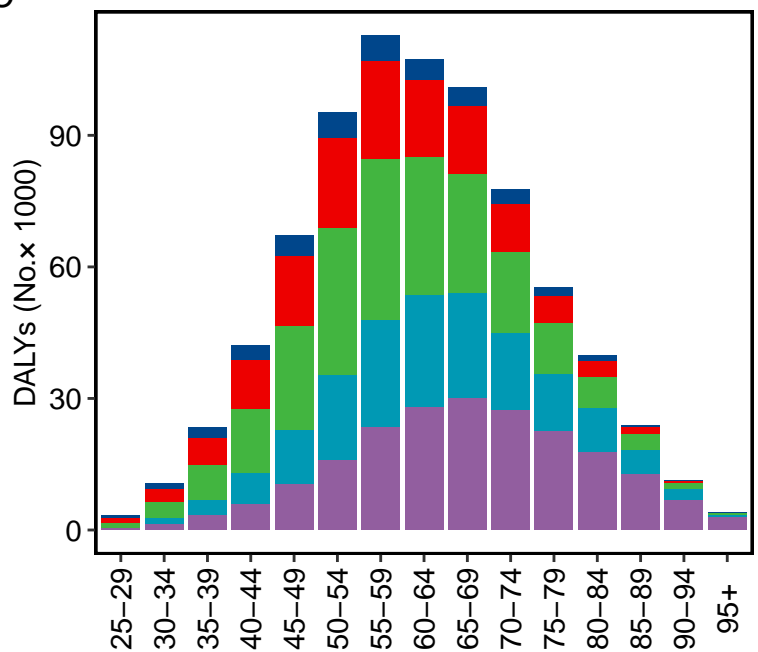

D

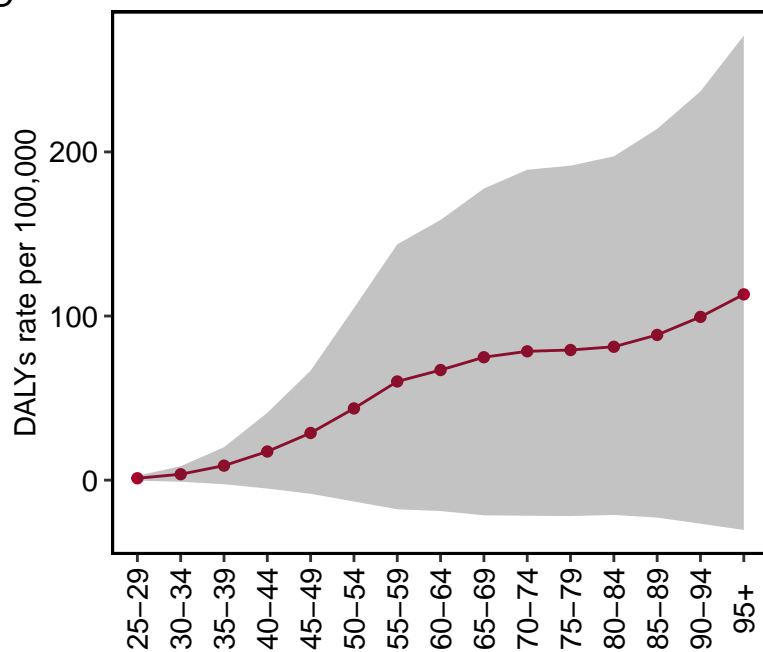

Low SDI Low-middle SDI Middle SDI High-middle SDI High SDI

Supplement: Supplementary Figure 3 — Breast cancer death (A) and DALYs (C) attributable to high fasting plasma glucose by age and SDI. The distribution of death (B) and DALYs (D) rate attributed to high fasting plasma glucose-related breast cancer by age. The grey area indicates the 95% uncertainty interval for the rate. DALYs, disability-adjusted life-years; SDI, socio-demographic index. [file Image3.pdf]

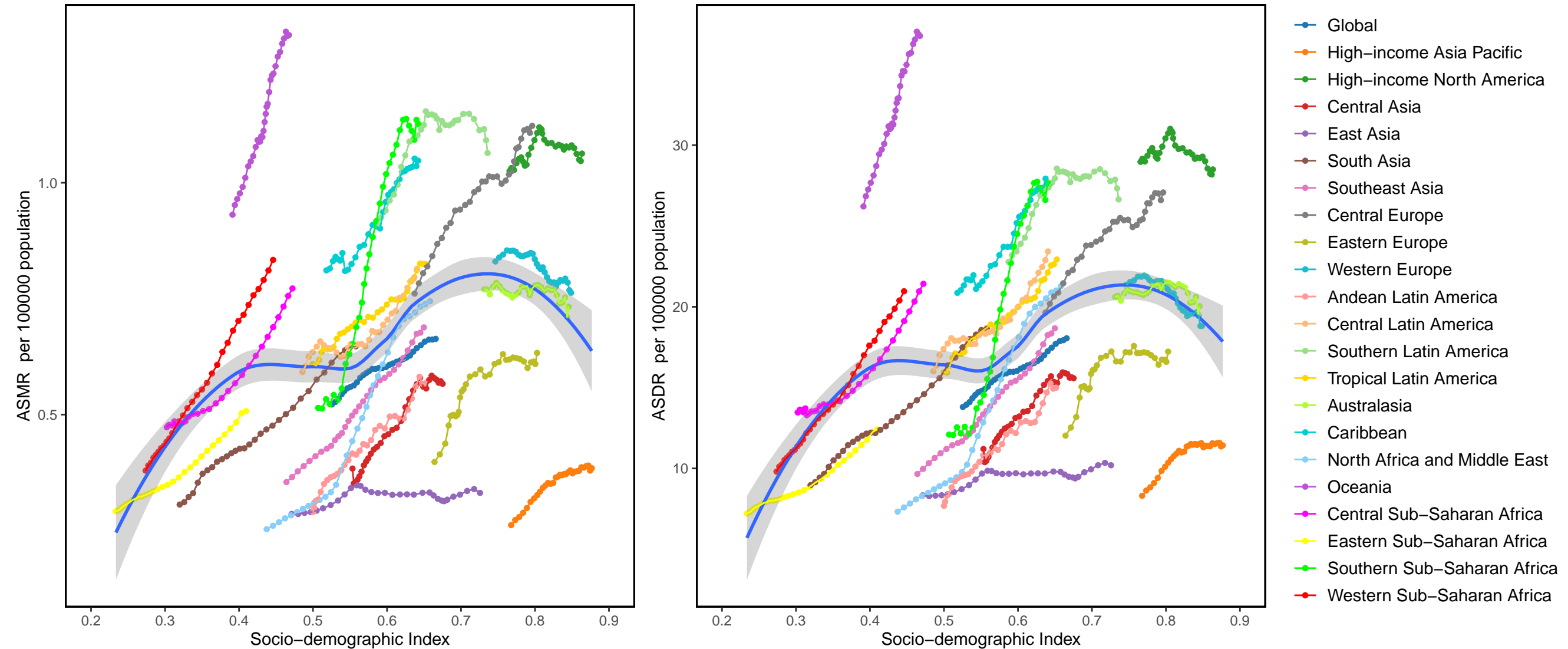

Supplement: Supplementary Figure 4 — The relationship between SDI and age-standardized rates (ASMR and ASDR) across 21 Global Burden of Disease regions. ASMR, age-standardized death rate; ASDR, age-standardized DALYs rate. [file Image4.pdf]

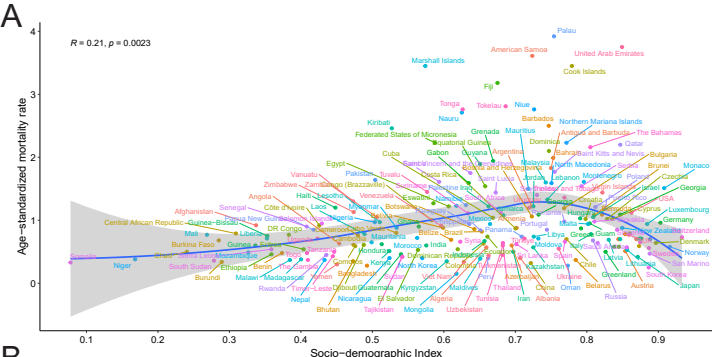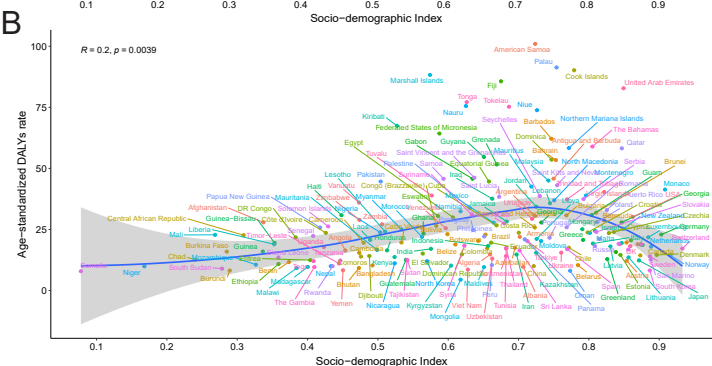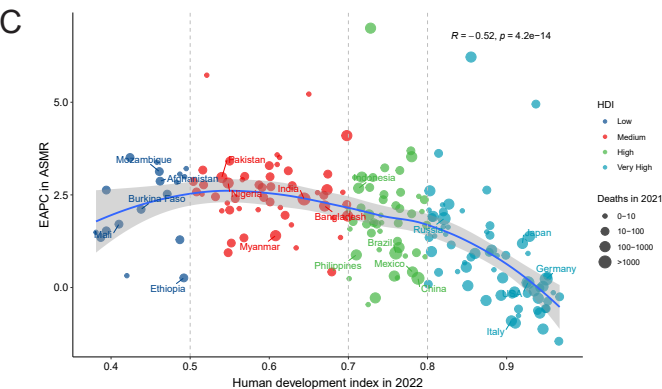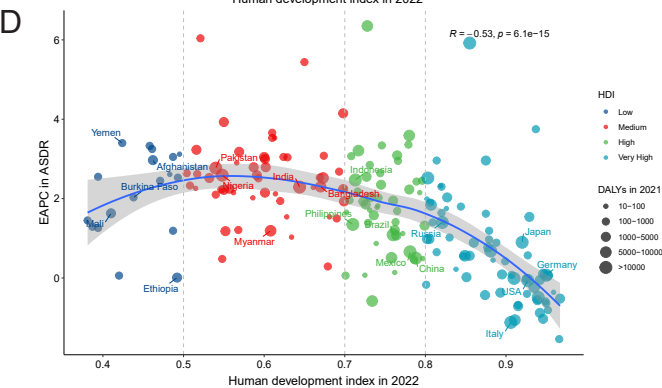

Supplement: Supplementary Figure 5 — The association between SDI and age-standardized rates for high fasting plasma glucose-related breast cancer across 204 countries or territories (A, B). The relationship between HDI and EAPCs of age-standardized rates for high fasting plasma glucose-related breast cancer (C, D). SDI, socio-demographic index; ASMR: age-standardized mortality rate; ASDR, age-standardized DALYs rate; EAPC, estimated annual percentage change; HDI, human development index. [file Image5.pdf]
